# Lessons learned from using high efficiency particulate air (HEPA) filtration units in care homes: interviews with care home staff, residents and relatives from the AFRI-c trial

**DOI:** 10.3310/nihropenres.13993.1

**Published:** 2026-07-31

**Authors:** Sophie Rees, Rachel Brierley, Clare Clement, Alastair Hay, Karen Sargent, Jane Sprackman, Ruth Kipping

**Affiliations:** 1 Bristol Trials Centre, University of Bristol Medical School, Bristol, England, UK; 2 Centre for Appearance Research, University of the West of England, Bristol, England, UK; 3 Centre for Academic Primary Care, University of Bristol Medical School, Bristol, England, UK; 4 Centre for Public Health, University of Bristol Medical School, Bristol, England, UK

**Keywords:** public health, respiratory infections, qualitative research, randomised controlled trial, HEPA filter, allergens

## Abstract

**Background:**

Respiratory infections are a leading cause of mortality and morbidity among older adults in residential care homes. We conducted a qualitative study within the ‘Air Filtration to prevent symptomatic winter Respiratory Infections (including COVID-19) in care homes (AFRI-c)’ pragmatic cluster randomised controlled trial. This paper reports the lessons learned from using high-efficiency particulate air (HEPA) filtration units in a care home setting.

**Methods:**

Interviews with care home staff (n=25), residents (n=20), and relatives (n=12) who participated in the AFRI-c trial were analysed using reflexive thematic analysis.

**Results:**

Preventing respiratory infections was a high priority for staff. Although using HEPA filters became normalised during the intervention period, they were not considered suitable for all residents. The ability to lock settings or fix them to walls was identified as a potential way to improve them in this setting. Participants felt that the care home sector would be unable to afford the cost of HEPA filters for all residents and that their use would be prioritised for those with the greatest need. However, without financial support, HEPA filters may only be used by residents who can afford them. Staff felt that HEPA filters might be attractive to potential residents and their families, but consultees and residents were both more focused on overall quality of care and life. Maintenance of the HEPA filters had a minor impact on staff workload.

**Conclusions:**

Although the AFRI-c trial found that HEPA filters did not reduce respiratory infections in residents, these insights could be valuable for care homes wishing to use HEPA filters for other reasons, such as reducing airborne pollutants and allergens. With some adaptions, portable HEPA filters could be made more suitable for use in care home settings. There were concerns that without financial support, inequalities could deepen.

## Plain English summary

Older people living in care homes are often vulnerable to respiratory infections (illnesses like flu, colds, and chest infections). High efficiency particle air (HEPA) filtration units, or ‘air filters’ can be used to filter the air and trap germs (like viruses) to stop them circulating in the air. This might stop people getting so many infections.

We did a large study where we put air filters in 91 care homes. About half (47) of the care homes that took part were given air filters and the other 44 care homes weren’t given air filters. We checked the number of days residents had colds, flu, or chest infections.

People with air filters had around the same number of days with illnesses as people without air filters.

As part of this study, we asked staff working at the care homes, residents, and also their relatives what they thought about the air filters. We took everything they said and analysed it to find patterns and understand what they thought.

Staff thought preventing infections was really important for the health of residents and the staff.

People had ideas for making the air filters more suitable to use in care homes.

Staff thought care homes probably wouldn’t be able to afford air filters without the government helping to pay for them. This could make things more unfair because people might only use them if they can afford them.

Staff also thought that having air filters might make a care home more appealing for people to move into, because they might think it is healthier. But residents and their families were more interested in how well residents were cared for in general, such as how attentive the staff were.

Having air filters in the care homes did not create a lot more work for the staff.

## Introduction

A leading cause of morbidity and mortality for older people living in residential care settings is infection with respiratory viruses, such as influenza and SARS-Cov-2. ^1, 2^ The interaction between care home residents, staff, and visitors makes it challenging to address respiratory infections, which can spread through both direct contact and airborne transmission. ^3^ There is potential to reduce the spread of respiratory viruses by using high-efficiency particulate air (HEPA) filters. These are known to remove airborne microbes, ^4^ but there is limited evidence about whether this results in fewer respiratory infections. ^5, 6^

The Air Filtration to prevent symptomatic winter Respiratory Infections (including COVID-19) in care homes (AFRI-c) randomised controlled trial (RCT) investigated the clinical- and cost-effectiveness of portable high efficiency particulate air (HEPA) filters in resident and communal rooms to reduce respiratory infection episodes in older residents. ^7^ The AFRI-c RCT included interviews as part of a mixed-methods process evaluation. Findings about acceptability, implementation, and fidelity of the use of HEPA filters are published elsewhere. ^8^

Introducing an intervention into a care home setting with vulnerable residents and busy staff, the useability, safety, and burden of the technology needs to be considered. ^9^ For example, there may be concerns about whether it will increase staff workload or present a safety risk (e.g., falls) in residents. Previous work assessing technologies to assist residents with dementia or cognitive impairment has called for research which includes understanding users’ perspectives on usability and acceptability. ^10^

The AFRI-c study found no evidence that infection episodes were reduced by using HEPA filters, ^7^ and therefore we are not recommending that the care home sector invest in this technology. Instead, we recommend that care homes focus on their routine infection prevention and control strategies and vaccination programmes. Nevertheless, some private contractors may wish to implement HEPA filters as manufacturers still report their efficacy in reducing particulate matter or removing allergens. ^11^ These possibilities, as well as potential future improvements in HEPA filters, may warrant research in the future. Therefore, we present lessons learnt from the use of portable HEPA filters in care homes, suggestions for modifications, views on funding, and staff workload. These findings may be beneficial for any future research investigating, or implementation of, HEPA filters in care homes.

## Methods

AFRI-c was a cluster RCT that took place in 91 care homes in England over three winters between September 2021 and May 2024. Each care home participated in a single winter period. Homes were randomised to either the use of HEPA filters (n=47) or usual care (n=44). The intervention care homes received portable HEPA filters for use in up to five communal rooms and between 10–16 bedrooms for their winter period. We used Philips™ HEPA filtration units: AC3033 for communal rooms and AC2936/33 for private bedrooms. These are freestanding units, each containing a filter consisting of three layers. Larger visible particles are captured in the outer layer, and a ‘brush’ light displays when it requires cleaning (vacuuming). The two self-contained inner layers capture particles >3 mm, and do not ‘leak’ viruses or bacteria during vacuuming. One of the reasons these units were chosen was that they are relatively robust and unlikely to be easily tipped over, which was seen as important in our discussions with care homes and Patient and Public Involvement (PPI) representatives. Care homes were asked to place the HEPA filters according to the manufacturer’s guidance, that is, >20 cm away from walls or objects and away from externally opening doors and windows. Staff were asked to keep the HEPA filters on sleep mode in bedrooms or ‘mode one’ in communal rooms. They were also asked to perform daily checks on the HEPA filters to check whether they were switched on and in the correct position. The full trial protocol provides further details. ^7^ The trial is registered in the ISRCTN (ISRCTN63437172) and commenced on 13/01/2022 and finished on 31/03/2025.

The AFRI-c process evaluation aimed to understand the acceptability, fidelity, and implementation of the AFRI-c intervention and contribute to the interpretation of clinical findings. The process evaluation was mixed methods, using interviews and surveys of multiple stakeholders. ^8^ In this paper, we draw solely on data from the qualitative interviews.

### Patient and Public Involvement (PPI)

When developing the study we formally convened an advisory group of adults who were either residents in a care home, work in a care home, or are family members of residents. Their feedback informed the study design including acceptable methods of contact for this population, length of case report forms and questionnaires, content in the resident information sheet, and logistics of consent models. Two members of that group (co-authors KS and JS) joined our team of co-investigators and we worked in partnership for the development and refinement of the resident and relative interview topic guides to ensure the appropriateness of the participant cohorts. For instance, they suggested that we changed some questions to focus more strongly on the present to avoid causing residents to feel frustrated or distressed if they could not remember something clearly. They also recommended breaking down the residents’ topic guide into higher- and lower-priority questions in case residents become tired throughout an interview. Our PPI members have also been involved in dissemination, such as contributing to the development of videos explaining the study, and posters to be distributed to the care home community.

### Participants

Participants in the qualitative study were care home staff (n=25) involved in delivering AFRI-c, residents (with capacity to provide consent for the research, n=20), and relatives (n=12) who had acted as personal consultees for a resident without capacity to consent. We only interviewed residents and relatives from the intervention care homes where the resident had a HEPA filter in their private room, as the focus was on their views/experiences of the intervention. We interviewed staff from both intervention and control care homes, as we also wanted to understand their views of delivering the trial.

### Recruitment and sampling

We used a mixture of convenience sampling (Winter 1) and maximum variation purposive sampling (Winters 2 and 3). To sample staff, we aimed for diversity in terms of: size of care home; regional location; and Index of Multiple Deprivation (IMD). To sample residents, we identified sites with high numbers of residents with both capacity to consent and who had agreed to be approached about the interview, sampling for variation in the IMD of the care home. For residents without capacity to consent, we sampled relatives for (resident) age, gender, and ethnicity.

We used study records to identify and approach study champions, inviting them to participate or to identify the most appropriate staff member(s) at the home to interview. To sample relatives, we used a report generated from the study database, first checking with care home staff about appropriateness to approach (e.g. relative had not recently transferred to hospital, deteriorated, or passed away). They were then approached via email and/or telephone, depending upon their stated preference. Residents were identified from the study database report, and the interviewer (SR) arranged a visit well in advance, checking upon arrival with the staff that it was appropriate to approach each named resident that day.

The relevant Participant Information Leaflet (PIL) was provided by email to staff and consultees by SR. Staff at the care home provided the PIL to residents prior to the scheduled visit and were asked to ensure someone had gone through the information with residents before the visit, and that they understood we were visiting.

### Data collection

Written informed consent was obtained for the AFRI-c trial and collection of quantitative data. We used telephone or video call interviews for care home staff and relatives, with verbal consent recorded at the start of each interview. Verbal consent was also audio-recorded for resident interviews, which all took place in person at the care homes, usually in the resident’s private room. Verbal consent was used for the qualitative interviews to reduce the burden on participants who had already given written consent to join the AFRI-c trial.

All interviews were conducted by SR (except for one staff interview conducted by CC). SR is a researcher, not a care worker, and although part of the trial team, participants were informed that SR was not involved in the design of the study, and they were invited to share their honest opinions. The interviews were audio-recorded and transcribed verbatim. Flexible topic guides relevant to the different participant groups were used. In Winter 3, the topic guides were less focused on trial and recruitment procedures and more on acceptability of the intervention.

### Analysis

We used reflexive thematic analysis ^12, 13^ to analyse the data supported by NVivo QSR. CC and SR independently coded a sample of transcripts and discussed developing codes and themes. We developed a flexible coding frame for coding the remaining transcripts, with the interpretation evolving with analysis. Continuous discussions between the team helped develop the analysis.

### Ethical approval

Ethical approval was given by London Harrow NHS Research Ethics Committee (Ref 21/HRA/4318). This research was carried out in accordance with the principles in the Declaration of Helskinki. ^14^

## Results

### Participants

We interviewed 57 participants from 22 care homes. Table 1 contains the characteristics of *care homes* in the qualitative study (n= 22) and *participant* (n=57) characteristics are in Table 2. Qualitative data collection took place during: March–May 2022 (Winter 1), November 2022–April 2023 (Winter 2), and February–April 2024 (Winter 3).

**Table 1.** Characteristics of care homes included in the qualitative study.

Care home characteristic	Number (%)
IMD decile * 1–5 6–10 *Mean*	10 (45%) 12 (55%) *6*
Region of England South West South East West Midlands North East North West East of England	9 (41%) 2 (9%) 4 (18%) 3 (14%) 1 (4%) 3 (14%)
Size (maximum resident capacity) 20–40 41–60 61+ *Mean size* *Range*	14 (64%) 5 (22%) 3 (14%) *41* *25–73*
Care Quality Commission (CQC)±rating (at baseline) Good Outstanding	21 (95%) 1 (5%)
Nursing care offered Yes	8 (36%)
Dementia care offered Yes	6 (27%)
Part of a chain of care homes Yes	10 (45%)
Trial arm Intervention Control	17 (77%) 5 (23%)
Winter 1 2 3	4 (18%) 10 (46%) 8 (36%)
**TOTAL**	**22**

* Index of Multiple Deprivation score, 1=most deprived, 10=least deprived; ±Only care homes with a CQC rating of Good or Outstanding were eligible for AFRI-c.

**Table 2.** Demographics of interviewed participants.

Participant	Characteristic	Number (%)
**Staff**	Role Manager Deputy Manager Carer/Senior Carer Activities Manager	13 (52%) 6 (24%) 5 (20%) 1 (6%)
	Length of experience 1–5 years 6–10 years 11–15 years 16–20 years >20 years	6 (24%) 2 (8%) 3 (12%) 2 (8%) 12 (48%)
	Nurse/carer background Yes	20 (80%)
	Care home providing nursing care Yes	10 (40%)
	Winter 1 2 3	7 (28%) 11 (44%) 7 (28%)
	Study arm Intervention Usual care	18 (72%) 7 (28%)
	**Total**	**25 (49% of all interviewees)**
**Residents**	Age 50–59 60–69 70–79 80–89 90–99 *Mean age*	1 (5%) 1 (5%) 4 (20%) 4 (20%) 10 (50%) *85*
	Gender Female Male	13 (65%) 7 (35%)
	Ethnicity White	20 (100%)
	Type of care Residential Nursing care	19 (95%) 1 (5%)
	Winter 1 2 3	0 (0) 11 (55%) 9 (45%)
	**Total**	**20 (35% of all interviewees)**
**Consultees**	Relationship to resident Daughter Daughter-in-law Son Stepdaughter Wife	1 (8%) 1 (8%) 5 (42%) 2 (17%) 3 (25%)
	Gender Female Male	7 (58%) 5 (42%)
	Ethnicity of resident White	12 (100%)
	Resident in nursing care Yes	7 (58%)
	Winter 1 2 3	4 (33%) 2 (17%) 6 (50%)
	**Total**	**12 (21% of all interviewees)**
**ALL**	**TOTAL**	**57**

### Alignment with health protection

Participants thought use of HEPA filters clearly aligned with care home priorities of protecting residents and staff and providing good care, which motivated people to participate in the trial.

*I’m always up for finding new ways of caring for elderly persons, for caring for others. Being a nurse it’s very important for me to be able to provide that best care … with COVID around and everything. –* 008, Winter (W)1 Staff, Intervention, Site D
*Anything that’s going to have an impact on reducing staff absenteeism as well, is a benefit to us. –* 039, W2 Staff, Intervention, Site N
*I felt it was in her* [relative’s] *interest that anything that’s trying to prevent and control infection and* [protect] *the people that look after her. Without them, the home would be shut, so selfishly, we need to keep everybody in the team safe and healthy because otherwise, there would be no care homes.* – 044, W3 Relative, Site P

The impact of the COVID-19 pandemic on care home residents and care homes was also mentioned as a specific reason for interest in participating in the HEPA filter trial.

*I think just coming out of the pandemic and everything I think it’s vitally really important that we look at ways that we can stop what happened the first time round happening again, should we end up in the same situation with regards to having to close care homes and things like that. It was devastating. –* 015, W2 Staff, Usual care, Site E
*I think one of the things with us at the moment, nursing homes are still being left behind. People are getting on with their normal lives … Even now people are still dying with it. People don’t realise …We’re still having to test our visitors on a weekly basis and ourselves …So if it can stop things like that happen again it would be of benefit. –* 017, W2 Staff, Usual care, Site F

### Views on improvements for HEPA filters

Elsewhere we have reported that the acceptability of the portable HEPA filters was high, and their use became normalised. ^8^ Despite this, there were a number of ways that staff suggested the use of the filters could be improved to fit better with the care home setting.

A key issue identified was that the HEPA filters were not suitable for care home residents with cognitive impairments who are mobile, both because they present a trip hazard and because residents might be confused and tamper with them.

*With nursing homes, you have people that wander around, that they might stick things in them. So again it’s like everything isn’t it, it’s just health and safety issues then …Is there a possibility instead of having stand based ones, would there be a possibility of having ones that go on the walls? Smaller versions.* – 017, W2 Staff, Usual care, Site F
*Not every client is going to be able to have them in their rooms and leave them alon*e *. They would [need] to be modified to fit to work in a dementia care home.* – 049, W3 Staff, Intervention, Site T

Another suggested modification was an ability to ‘lock’ the settings or have a lockable cover over the buttons so that settings cannot be changed by residents or staff.

*It’s like a digital touchscreen. That could do with a cover which would, then, stop people fiddling with it …Night staff do cleaning at night in the communal areas, and [name] goes round and checks in the morning ‘cause they put ‘em on ‘Sleep mode’...They’ve just, obviously, wiped across the top of it, and pressed ‘Sleep mode.’* – 034, W2 Staff, Intervention, Site K
*Clients can just put their hand on them and they could change the settings …Because people walk past them or whatever, they’ve only got to touch them with their hand and they were changing settings. Well, they still do, obviously. So, yeah, I think if they had some sort of lock on it, just a PIN number to go in to look at them.* – 049, W3 Staff, Intervention, Site T

### Views on adoption of HEPA filters

Most people thought that if the HEPA filters were effective, then they should be implemented. However, staff felt that few homes would adopt them unless there was very strong evidence and there were grants available for funding. Our study care homes received a mixture of private, charitable, and local authority funding. The financial constraints of social care and the care sector means that there would be issues of prioritisation and affordability if the filters were to be used beyond the research study.

*We’re a registered charity and money’s tight in social care, ridiculously tight at the moment, so there’s no way it would feature on our high list of priorities.* – 018, W2 Staff, Usual care, Site G
*Unless it’s proven that it’s extremely helpful and everything and I mean the findings would be beneficial and everything, I don’t think every home in the area, or maybe across the UK will go with them. Mainly from a financial point of view.* – 008, W1 Staff, Intervention, Site D
*With the cost of everything going up, food, gas, electric, water has gone up. A lot of care homes are literally just breaking even, so I can't see them doing that any time soon. If prices start to come down and it was proved that these are able to reduce infections and that, I think a lot of smaller care homes would probably consider it, but the bigger care homes probably wouldn’t because of the scale.* – 039, W2 Staff, Intervention, Site N

Care home staff and relatives thought government funding should be provided to pay for HEPA filters, if they had been found to be effective, through grants for capital costs but there were also concerns about revenue expenditure for the energy and maintenance costs.

*I would say yes, if they're found to be useful then they should be in in all care homes, why not? But then it always comes down to who should pay, and the reality is that care is very short on money …As it stands at the moment if there was a situation where they said, ‘Okay, well the government will do 50%,’ or whatever, ‘but then the care homes have to stump up the rest,’ I'm not convinced the care homes would in all honesty, not because they wouldn't want it, but finances.* – 046, W3 Relative, Site S
*Even if the government paid for the air filters, it’s the cost of running. I know it’s probably a minimal cost but it’s still the cost of running that would go onto the care homes. With fuel bills like they are, I doubt some care home owners would entertain them, to be fair …I think that our owners would want a lot of proof that they’re working.* – 049, W3 Staff, Intervention, Site T

### Prioritising use based on greatest need

Given the challenge of costs of HEPA filters for every room, some participants proposed that HEPA filters could be prioritised according to who would most benefit, for example, those with underlying respiratory conditions.

*It might be something if we’ve got residents who’ve got long-term conditions like COPD, they might want to do that …For example, in a resident who like I say has got COPD, any sort of extreme asthma or anything like that, if that would help them.* – 020, W2 Staff, Usual care, Site H
*Someone who suffers with COPD, could have it prescribed by their GP for example. Or asthma clinics can prescribe them for the vulnerable, to start with. A prescription. Like they can prescribe you, I don’t know, to go swimming.* – 060, W3 Staff, Intervention, Site V

At least one site was planning to install HEPA filters but prioritise them for certain residents.

*We’ve already spoke to a director about it. He is in agreement. He’s been looking at prices of the air filters. He’s quite happy, paying for the air filters himself. It’s just putting them in the bedrooms if people spend most of the time in their room …People who are more prone to infections, the people who obviously get a lot of chest infections and have a lot of coughs and colds over the year. I think they would probably prioritise them.* – 003, W1 Staff, Intervention, Site A

### Health inequalities

There was a recognition that without financial support for homes to install them, filters would likely be used by individuals with the means to purchase them, which could increase health inequalities.

*Is it going to be the privileged few, obviously with the costs and that. We have over 55 residents, so would we be able to fund one for every room within the home for each individual resident?.*.. *Not everybody is in the home has relatives that have that kind [of money].* – 017, W2 Staff, Usual care, Site F
*If it’s going to make a vast difference then it should be backed I would have thought by the government and the NHS because it’s going to help everybody in the long run isn’t it. It can’t be down to those who can afford it, it needs to be across the board.* – 061, W3 Staff, Intervention, Site V

### Attracting potential residents

Some staff suggested that having HEPA filters might make the care home more appealing to potential residents.

*I think if I was going to look at a care home for my mum and one of the care homes had the air filters I think I’d be a little bit more swung to that, because I’d be thinking, well they’re really thinking about the residents here and preventing infections and infection control etc.* – 016, W2 Staff, Usual care, Site E

When we asked relatives about this, one said that they would prefer a care home that had HEPA filters if they were effective, but others felt it would have less impact than the overall feel of a care home and their quality of care.

*Between a home that had filters and didn’t, and there wasn't an awful lot between them in every other aspect, I’d probably go for the one with filters. I guess if there's more information to back up the benefits of filters then that becomes your decision then or part of your decision-making …I think you know, you’re prepared to pay a little bit more to make sure your parents are well looked after, yes, and just hope the money lasts.* – 007, W1 Relative, Site B
*I think the quality of care is more important …I did go and look at lots of care homes and lots of them had lots of facilities which looked very nice and I thought if that was me going in there, yes, fine, that would be great, it would be lovely to have a cinema, a bar, a hairdressers and everything but at the end of the day it’s the quality of the care and are the staff caring, and is he looked after, and is it clean, and is it a healthy environment for him to be in, which are more important. So I think depending on the cost I think if it wasn’t that much different then if I thought they were working and that had been proved in the study they had yeah, I would probably go for one but I think overall from that is the quality of care that the home is offering.* – 043, W3 Relative

Generally, residents also did not highly value the introduction of HEPA filters and focused more on the quality of care and attentiveness of the staff.

*I’m happy here in this place to tell you the truth. People say a lot of care homes and that they’re horrible but good gracious me they can’t do enough for you. They help me. It’s getting dark, I say ‘can you pull the curtains over’ and they come and pull the curtains over. And I’ve got this [alarm] and I’ve got this [gestures] I’m quite happy here. I wouldn’t fault anything …I’m quite happy here, with my lot.* – 024, W2 Resident, Site J

### Staff workload

There was an awareness of the ongoing staff time required to clean and maintain the filters. However, we found that the HEPA filters were embedded without issue into the routine workload, and if rolled out this was expected to be part of domestic staff workload.

*a bigger filter that needs less maintenance …Or even something that’s got automation on there, that automatically cleans filters, or something …I think the bigger you go and the bigger the filter the less maintenance you probably have to do on it. I don’t know whether that’s right, I’ve no idea.* – 060, W3 Staff, Intervention, Site V
*I think if they were in every room, it would probably become part of the domestics’ job, who go in there every day with the hoover, so they’d clean the filters when they came up anyway. No, I don’t think it would. It’s not a great inconvenience at all …I think if the results from the study are conclusive then I think there’s no reason why you wouldn’t want to do that, would you, you know? –* 045, W3 Staff, Intervention, Site R
*I don’t think it would be particularly bad. It* [cleaning/checking filters] *would just be another sort of job that's built into the routine, I suppose.* – 055, W3 Staff, Intervention, Site U

## Discussion

Staff and relatives felt that the use of HEPA filters aligned with their priorities for protecting the health of residents and staff. The COVID-19 lockdowns were also a motivator to reduce the impact of infections and avoid similar experiences. Some staff members thought that the presence of HEPA filters might attract more residents. However, relatives valued quality of care over the use of technological interventions, such as HEPA filters. We have reported elsewhere that preventing infections was not necessarily a high priority for all residents, who were more concerned with overall quality of life and care. ^8^ Therefore, any future implementation of HEPA filters should include careful consideration of residents’ values and wishes if their purpose is to reduce infections.

Generally, a technology will have higher acceptability if it has good usability (when a technology or device can be easily and effectively used by humans) and can be adapted to the needs and preferences of the group or setting of interest. ^9, 15^ The HEPA filters were generally considered to have good usability, but there were a number of suggestions participants made that could improve the appropriateness of HEPA filters to care homes. These included being able to lock the settings, or being able to mount them to the wall to avoid trip hazards and avoid cluttering private bedrooms. The maintenance of the HEPA filters was not considered onerous, and it was felt by staff that it could be easily incorporated into the work of domestic staff.

Most participants felt that the financial realities of the care home sector meant that widespread adoption of HEPA filters was unlikely, even if they were found to be effective, without government assistance. The HEPA filters used in AFRI-c cost approximately £260 each, electricity running costs are estimated at around £55 a year, and the internal filter needs yearly replacement, costing £62 each. ^16^ Some participants were concerned that without government support, HEPA filters might only be used by those homes or individual residents who could afford to purchase them, which would deepen health inequalities. Although we are not recommending that HEPA filters are adopted by the care home sector, because we found they did not reduce infections, this concern applies to other preventative interventions which could be used selectively for some care home residents based on ability to pay.

### Strengths and limitations

A qualitative approach enabled us to explore views and experiences in depth and identify issues important to participants. The sample size (n=57) is relatively large for a qualitative study, but still only represents a small proportion of the large numbers of staff, residents, and relatives who were involved with the AFRI-c study. Individual interviews facilitated participants to express a wide range of views, including some that might not appear socially acceptable. An observational or ethnographic approach may have identified challenges with using the HEPA filters which participants did not mention in interviews. Participants were aware that the researcher had no competing interests about the HEPA filters’ acceptability. We were able to look at the same topic from the perspective of three cohorts (staff, residents, relatives) who may have different priorities.

## Conclusions

Although we are not recommending that care home adopt HEPA filters to reduce respiratory infections, the lessons learned here may be valuable for future researchers, or for stakeholders wishing to adopt HEPA filter use to reduce airborne pollutants or allergens in care homes. If introducing or evaluating HEPA filters in the care home setting, adaptations such as the ability to lock the settings, and affix them to the wall could make them more suitable. The HEPA filter maintenance for the air filters used in AFRI-c did not significantly increase workload and could be embedded into routine practice easily. Staff may believe that the use of technology such as HEPA filters will attract residents, but families are more concerned with the overall quality of life and care they/their relative will receive in the home. Care home staff are concerned that preventative technologies could exacerbate health inequalities if government financial support is not available. 

## Ethical approval

Ethical approval was given by London Harrow NHS Research Ethics Committee (Ref 21/HRA/4318).

## Consent

Written informed consent was obtained for the AFRI-c trial. For the qualitative interviews, verbal (audio-recorded) consent, including for the publication of participant data was obtained from all participants. Verbal consent was chosen to reduce the burden on participants who had already given written consent to join the AFRI-c trial.

## Data Availability

Data for this study are not available on an open licence because this would breach the protocol approved by the research ethics committee and the consent provided by participants. However, these data are published as a restricted dataset at the University of Bristol Research Data Repository data.bris, at https://doi.org/10.5523/bris.250w5vo8i6he32dc6y3plrq8d9. The metadata record published openly by the repository at this location clearly states how data can be accessed by bona fide researchers. Requests for access will be considered by the University of Bristol Research Data Service, who will assess the motives of potential data re-users before deciding to grant access to the data. No authentic request for access will be refused and re-users will not be charged for any part of this process. ^17^ ‘Extended data’ (topic guides, information sheets, consent forms) are available unrestricted from: https://doi.org/10.5523/bris.106jsm1tvvsg62gf2d1tofsh57. Data are available under the terms of the Creative Commons Attribution 4.0 International license (CC-BY 4.0). ^18^
