# Supplementary material for: Lessons learned from using high efficiency particulate air (HEPA) filtration units in care homes: interviews with care home staff, residents and relatives from the AFRI-c trial
Source: NIHR Open Res. 2026 Oct 6;6:111. Originally published 2026 Jul 31. [Version 2] doi: 10.3310/nihropenres.13993.2 (PMC13631442; doi:10.3310/nihropenres.13993.2)
Supplement: Peer review history

# Reviewer report: Reviewer response for version 2

**DOI:** 10.3310/nihropenres.15781.r42776

**Published:** 2026-10-08

**Authors:** Julii Brainard

**Affiliations:** 1 University of East Anglia, Norwich, England, UK

I have no further comments except to say thanks for making to put your trial results and the lessons learned into the public domain; so many trials with "negative" results never get fully described.

Is the work clearly and accurately presented and does it cite the current literature?

Partly

If applicable, is the statistical analysis and its interpretation appropriate?

Not applicable

Are all the source data underlying the results available to ensure full reproducibility?

Partly

Is the study design appropriate and is the work technically sound?

Yes

Are the conclusions drawn adequately supported by the results?

Yes

Are sufficient details of methods and analysis provided to allow replication by others?

Partly

Reviewer Expertise:

Systematic reviews; epidemiology; qualitative synthesis; infectious diseases.

I confirm that I have read this submission and believe that I have an appropriate level of expertise to confirm that it is of an acceptable scientific standard.

# Reviewer report: Reviewer response for version 1

**DOI:** 10.3310/nihropenres.15213.r41964

**Published:** 2026-10-01

**Authors:** Douglas Booker

**Affiliations:** 1 University of Leeds, Leeds, UK

The paper presents a mixed-methods process evaluation of a study understanding the efficacy of portable HEPA air cleaners in care homes, focusing on qualitative interviews with care home staff, relatives, and family members. I enjoyed reading the paper, which was very-well written, and easy to follow. I particularly enjoyed the strong public member involvement in the project (including co-authorship of the paper, and you clearly outline their involvement). Moreover, it is very helpful for other projects to see a qualitative reflection on a ‘technical’ intervention like air cleaners, which often only covers their effectiveness at reducing PM from the air and/or links to measurements of health.

 I have marked this as 'approved with reservations', which I would classify as minor. 

 The work is clearly and accurately presented, but there were a few occasions where I felt further literature would be helpful to support claims / position the work.

1. While characterising the efficacy of HEPA air cleaners is not the main purpose of this paper, I found these lines in the introduction as a bit of an oversimplification “The AFRI-c study found no evidence that infection episodes were reduced by using HEPA filters,7 and therefore we are not recommending that the care home sector invest in this technology. Instead, we recommend that care homes focus on their routine infection prevention and control strategies and vaccination programmes. Nevertheless, some private contractors may wish to implement HEPA filters as manufacturers still report their efficacy in reducing particulate matter or removing allergens.11” You found they didn’t influence it, but what about other studies, including in other settings (e.g. schools)? Could it be a problem with the design of your study? You looked at portable HEPA ai cleaners, rather than HEPA filters (e.g. which could be put into a HVAC system). I also do not think it is fair to say it is only manufacturers that report the efficacy of HEPA filters in removing PM etc. I think you cover these topics nicely in  a more nuanced way in the protocol paper that you reference (doi:10.1001/jamainternmed.2026.2199), so perhaps you could just signpost to that paper again here to show that you have that conversation in more depth elsewhere, e.g *“* *There is clear evidence that HEPA filters are capable of cleansing the air of pollutants,* *^22^*** *allergens,* *^23^*** *and microbes* *^14^* *^,^* *^24^* *; that they can improve quality of life* *^22^* *; and that clean air increases longevity.* *^25^*** *A small, individually randomized crossover trial found some possible signals of HEPA filter effectiveness in reducing respiratory infections in care home residents but appeared underpowered.* *^26^*** *The present trial is larger, randomized at the care-home level, and provides evidence consistent with 2 systematic reviews, which concluded that portable HEPA filters do not reduce respiratory infection acquisition in private homes and offices.* *^27^* *^,^* *^28^* *^”^*
2. I felt that the discussion would be strengthened if there was engagement / comparison with further literature that done a process-based evaluation of an air cleaner study. That could be in other care homes, or if that evidence is not available, settings with vulnerable people, such as schools?

With regards to the methods, there were a few points where I felt further explanation would help with clarity / replication:

1. “We only interviewed residents and relatives from the intervention care homes where the resident had a HEPA filter in their private room, as the focus was on their views/experiences of the intervention. We interviewed staff from both intervention and control care homes, as we also wanted to understand their views of delivering the trial.” - You mention that you interviewed staff from both the intervention and control arms, as you wanted to understand their views of delivering the trial. I could not quite work out how the views of those in the control arm would be helpful for a process-based evaluation as they had not received the intervention? Perhaps you can further clarify this?
2. “We used reflexive thematic analysis12,13 to analyse the data supported by NVivo QSR. CC and SR independently coded a sample of transcripts and discussed developing codes and themes. We developed a flexible coding frame for coding the remaining transcripts, with the interpretation evolving with analysis. Continuous discussions between the team helped develop the analysis” – more information on your qualitative coding would be helpful. The principle of analysis is useful information: “interpretation evolving’ sounds like inductive coding approach? Or did you go in with some preset themes that you read the data against? Likewise, your specific method is helpful information. For example, did you do a line by line open coding, or something else? It would also be useful to know what themes emerged. I am guessing that these are broadly related to the subsections in the results (e.g. alignment with health protection, improvement/adoption of HEPA filters, prioritising use based on greatest need etc), but that could be clearer.

I marked the ‘source data underlying the results available to ensure full reproducibility’ as partly as it is available upon request, but this is a sensible approach.

Is the work clearly and accurately presented and does it cite the current literature?

Partly

If applicable, is the statistical analysis and its interpretation appropriate?

Not applicable

Are all the source data underlying the results available to ensure full reproducibility?

Partly

Is the study design appropriate and is the work technically sound?

Yes

Are the conclusions drawn adequately supported by the results?

Yes

Are sufficient details of methods and analysis provided to allow replication by others?

Partly

Reviewer Expertise:

Indoor air quality, qualitative methods, environmental justice, complex systems, air quality sensors

I confirm that I have read this submission and believe that I have an appropriate level of expertise to confirm that it is of an acceptable scientific standard, however I have significant reservations, as outlined above.

# Reviewer report: Reviewer response for version 1

**DOI:** 10.3310/nihropenres.15213.r41961

**Published:** 2026-09-02

**Authors:** Alice Turner

**Affiliations:** 1 University of Birmingham, Birmingham, England, UK

This report concerns the qualitative work package accompanying a trial of HEPA air filters in care homes which sought to reduce infections over winter. The purpose of this analysis was to explore how acceptable and practical the intervention would be. The authors should be congratulated on managing to interview a substantial number of people representing a range of residents, relatives and carers. In general the text is easy to read, methods are clear and conclusions supported by the results. Whilst the primary trial was negative such that routine recommendation is not supported by the authors, the lessons learned from this data about what stakeholders value in this setting and the likely issues for the health service are valid. Issues of funding for devices are highly relevant to raise, and the supportive quotes about routes for GPs to prescribe (eg comparing to exercise on prescription) are potentially important for policy makers. The literature cited is relevant to the field and the strengths and weaknesses are adequately described. The potential for devices to have benefits beyond infection is relevant but unable to be explored in detail in this manuscript; secondary or subgroup analyses of the main trial population might be able to explore this more fully.

Is the work clearly and accurately presented and does it cite the current literature?

Yes

If applicable, is the statistical analysis and its interpretation appropriate?

Not applicable

Are all the source data underlying the results available to ensure full reproducibility?

Partly

Is the study design appropriate and is the work technically sound?

Yes

Are the conclusions drawn adequately supported by the results?

Yes

Are sufficient details of methods and analysis provided to allow replication by others?

Yes

Reviewer Expertise:

I work in academic respiratory medicine and am familiar with indoor air quality studies.

I confirm that I have read this submission and believe that I have an appropriate level of expertise to confirm that it is of an acceptable scientific standard.

# Response

**Published:** 2026-09-25

**Authors:** Sophie Rees

**Affiliations:** Bristol Trials Centre, Bristol University, Bristol, UK

Thank you to this reviewer for their report and kind words. We refer this reviewer to our clinical results paper and Supplement 2 which report multiple pre-planned subgroup and secondary outcome analyses: 

 Hay AD, Brierley RCM, Turner N, et al. High-Efficiency Particulate Air Filters to Prevent Winter Respiratory Infections in Care Homes: The AFRI-c Cluster Randomized Clinical Trial. *JAMA Intern Med.* 2026;186(9):1124–1134. doi:10.1001/jamainternmed.2026.2199

# Reviewer report: Reviewer response for version 1

**DOI:** 10.3310/nihropenres.15213.r41711

**Published:** 2026-08-28

**Authors:** Julii Brainard

**Affiliations:** 1 University of East Anglia, Norwich, England, UK

OVERVIEW:

 This is useful and clearly written. 

 I was impressed by the pragmatism of the respondents in figuring out what they thought about the filters.

 My comments amount to quite minor revisions.

 With regard to data availability I graded this as partly because the data are supplied upon request (which is entirely reasonable and appropriate) rather than immediately available. 

 Some additional references are suggested (hence “partly” on the grading for citation of up to date literature).

 I graded the submission as “partly” for replicability because I was unable to access the supplemental data repository (“site not found”)

https://doi.org/10.5523/bris.250w5vo8i6he32dc6y3plrq8d9  (that link didn’t work; perhaps because this item is still unpublished).

 All of those things should be acceptable (data availability) or easily fixed before indexing (additional refs and access to documents like the topic guides).

 On the manuscript itself,

 I was struck by the lack of description about cost-effectiveness results.  Cost-effectiveness was part of the original protocol.  Presumably because of the lack of difference between groups in infection rates, no formal CE comparison was undertaken.  But then the article does talk about capital and running cost of the HEPA units, and needing “strong” evidence in favour of their use for them to be widely adopted on cost grounds, and thus unnecessary/unprotective expenditure becomes very relevant.   It seemed odd to not have at least a sentence saying something that the units as deployed in this experiment were inherently not cost-effective for infection prevention and quite possibly not even cost-effective to prevent asthma attacks or COPD exacerbation, see results in this syst. Review for instance: https://pmc.ncbi.nlm.nih.gov/articles/PMC13064970/

 There is a good-looking protocol for syst review about asthma prevention using air filters; I can’t find a published version of the results but I would (email those review protocol authors to ask if they have published because I would) want to cite that review’s results in this ”lessons learned” Afri-c article. https://bmjopen.bmj.com/content/14/12/e087493.abstract

 Methods: please explain what is “Mode one” ?  Low rate of air flow, maybe?

 … what are “study champions”?  What role did they have in general?

 References (even just one Ref for each of next 2 points) could be added to further describe …

 …what is maximum variation purposive sampling

 …what is IMD, which is presumably 2019 version

 The sentence that ends

 “someone had gone through the information with residents before the visit, and that they understood we were visiting.”

 Should that say

 “someone had gone through the information with residents before the visit, and that they understood **why** we were visiting.”

 RESULTS, I wondered if 57 participants were “from” care homes or “associated with” the care homes

 OTHER COMMENTS

 The PPI was well explained and sounded well-incorporated; exemplary really.

Is the work clearly and accurately presented and does it cite the current literature?

Partly

If applicable, is the statistical analysis and its interpretation appropriate?

Not applicable

Are all the source data underlying the results available to ensure full reproducibility?

Partly

Is the study design appropriate and is the work technically sound?

Yes

Are the conclusions drawn adequately supported by the results?

Yes

Are sufficient details of methods and analysis provided to allow replication by others?

Partly

Reviewer Expertise:

Systematic reviews; epidemiology; qualitative synthesis; infectious diseases.

I confirm that I have read this submission and believe that I have an appropriate level of expertise to confirm that it is of an acceptable scientific standard, however I have significant reservations, as outlined above.

# Response

**Published:** 2026-09-25

**Authors:** Sophie Rees

**Affiliations:** Bristol Trials Centre, Bristol University, Bristol, UK

**Reviewer 1**

 This is useful and clearly written. I was impressed by the pragmatism of the respondents in figuring out what they thought about the filters. My comments amount to quite minor revisions. Some additional references are suggested (hence “partly” on the grading for citation of up to date literature).

 With regard to data availability I graded this as partly because the data are supplied upon request (which is entirely reasonable and appropriate) rather than immediately available. I graded the submission as “partly” for replicability because I was unable to access the supplemental data repository (“site not found”) https://doi.org/10.5523/bris.250w5vo8i6he32dc6y3plrq8d9  (that link didn’t work; perhaps because this item is still unpublished).

***Response: We have checked and confirmed that this link is now working.***

 All of those things should be acceptable (data availability) or easily fixed before indexing (additional refs and access to documents like the topic guides).

 On the manuscript itself,

 I was struck by the lack of description about cost-effectiveness results.  Cost-effectiveness was part of the original protocol.  Presumably because of the lack of difference between groups in infection rates, no formal CE comparison was undertaken.  But then the article does talk about capital and running cost of the HEPA units, and needing “strong” evidence in favour of their use for them to be widely adopted on cost grounds, and thus unnecessary/unprotective expenditure becomes very relevant.   It seemed odd to not have at least a sentence saying something that the units as deployed in this experiment were inherently not cost-effective for infection prevention and quite possibly not even cost-effective to prevent asthma attacks or COPD exacerbation, see results in this syst. Review for instance: https://pmc.ncbi.nlm.nih.gov/articles/PMC13064970/

***Response: While we appreciate the reviewer’s point, our cost-effectiveness results are due to be published elsewhere, and our clinical results are already published. We have intentionally avoided replicating discussion of clinical- and cost-effectiveness results to keep this article focused on useful lessons learned for future research or other circumstances where air filters may be used in the residential care setting.***

 There is a good-looking protocol for syst review about asthma prevention using air filters; I can’t find a published version of the results but I would (email those review protocol authors to ask if they have published because I would) want to cite that review’s results in this ”lessons learned” Afri-c article. https://bmjopen.bmj.com/content/14/12/e087493.abstract

***Response: As above – an interesting article but discussion of clinical/cost effectiveness is beyond the scope of this article.***

 Methods: please explain what is “Mode one” ?  Low rate of air flow, maybe?

 … what are “study champions”?  What role did they have in general?

***Response: We have added further description of these points.***

 References (even just one Ref for each of next 2 points) could be added to further describe …

 …what is maximum variation purposive sampling

 …what is IMD, which is presumably 2019 version

***Response: References added.***

 The sentence that ends “someone had gone through the information with residents before the visit, and that they understood we were visiting.” Should that say “someone had gone through the information with residents before the visit, and that they understood why we were visiting.”

***Response: Thank you, amended.***

 RESULTS, I wondered if 57 participants were “from” care homes or “associated with” the care homes

***Response: Amended as per this suggestion.***

 OTHER COMMENTS

 The PPI was well explained and sounded well-incorporated; exemplary really.

***Response: We thank the reviewer for their kind comments on our article.***
